# Supplementary material for: Ecosystem service value evaluation method in a complex ecological environment: A case study of Gansu Province, China
Source: PLoS One. 2021 Feb 5;16(2):e0240272. doi: 10.1371/journal.pone.0240272 (PMC7864441; doi:10.1371/journal.pone.0240272)
Supplement: Attachment — Submitted filename: reviewers comments.docx [file pone.0240272.s002.docx]

comments to the Author:

The first time I looked at the title " Evaluation method of ecosystem service value under complex ecological environment: A case study of Gansu Province, China ", I was just wondering to know how would the authors deal with this topic. I was a little impressed and felt that this might be a very interesting manuscript. When I reviewed the manuscript, I didn't feel disappointed. In the manuscript, On the one hand, the average value equivalent factor per unit area of different ecosystems is determined in Gansu Province, with reference to the six calculation process. This method of meta-analysis will avoid or reduce the subjective conjecture which is easy to be caused by relying on experts' experience; On the other, through abundant eco-environmental data, this study established a value evaluation model and completed the evaluation of ecosystem service value under complex ecological environment. In particular, one thing is affirmed that the human activity factors that affect ecosystem service value are fully considered by Author to the evaluation of ecosystem service value, such as carrying capacity, air pollution, over exploitation of groundwater and water pollution etc. The methods and data used in the study are new. The manuscript meets the requirement for acceptation for publication.

My main concerns are as follows:

Introduction

1. The description of Gansu Province may be suitable for the part of study area.

2. There lacks enough literature review on the topic in the study area. The authors may check some references on this topic in last decades.

Materials and Methods

1. What is the basis of the ecosystem type classification?

2. Please supplement the distribution map of ecosystem types

3. from 2000-2015, what does this mean? The authors have to state clearly which year was used.

4. Classification of ecosystem service functions. Please explain the specific problem of repeatability. The description is not fully understood due to the ecological integrity has been evaluated in previous research.

Result

1. In the Table3, the unit of area is km^2^, while in formula 19, the unit of area is ha.

2. Biodiversity maintenance value regulation index (a11), How the habitat quality index is measured?

3. "invest" should be "InVEST"

Other detail question

English needs proofreading and editing, especially those sentences related to comparison. In addition, there exists many formatting problems in this manuscript, please verify the full text.

1.Please unify the number of decimal points in the whole text.
2. Table1. Please note that the black space.
