# Supplementary material for: Ecosystem service value evaluation method in a complex ecological environment: A case study of Gansu Province, China
Source: PLoS One. 2021 Feb 5;16(2):e0240272. doi: 10.1371/journal.pone.0240272 (PMC7864441; doi:10.1371/journal.pone.0240272)
Supplement: Attachment — Submitted filename: Response to Reviewers.docx [file pone.0240272.s003.docx]

Response to Reviewers

Reply to Reviewer #1

**Comment1:**

*“In introduction, It is recommended that authors add comparisons with other ecosystem service value studies and introduce the advantages of the approach used in this article.”*

**Response1:**

We added comparisons with other ecosystem service value studies and introduce the advantages of the approach used in this article, line108-118 in marked-up copy of my manuscript. The modified part is highlighted in yellow.

**Comment2:**

*“In the discussion section, I suggest comparing with other ecological service value assessment methods in Gansu Province.”*

**Response2:**

There are few methods to evaluate the value of ecosystem services in Gansu Province, through literature retrieval, we compared with the study by Wang et al; line735-736 in marked-up copy of my manuscript. The modified part is highlighted in yellow.

**Comment3:**

*“There are still some problems in English grammar and sentences. Please check and correct them carefully.”*

**Response3:**

We used Editage for English language editing that edited my manuscript, and we have carefully and thoroughly proofread the manuscript to correct all the grammar and typos. For details of manuscript editing, please refer to marked-up copy of my manuscript.

Reply to Reviewer #2

**Comment1:**

*“In introduction, the description of Gansu Province may be suitable for the part of study area.”*

**Response1:**

In introduction,“Gansu Province is located in the northwestern inland in China. …..Its special geographical location and natural conditions form a more distinct ecological structure.”, this part is repeated with the content in the study area, so this description of Gansu Province has been deleted in introduction. line 119 in marked-up copy of my manuscript. The modified part is highlighted in yellow.

**Comment2:**

*“There lacks enough literature review on the topic in the study area. The authors may check some references on this topic in last decades. ”*

**Response2:**

We checked some references about method of ecosystem services value, and added other ecosystem service value studies. line 108-118 in marked-up copy of my manuscript. The modified part is highlighted in yellow.

**Comment3:**

*“What is the basis of the ecosystem type classification? ”*

**Response3:**

The basis of the ecosystem type classification was added in Ecosystem type data, line 160-184 in marked-up copy of my manuscript. The modified part is highlighted in yellow.

**Comment4:**

*“Please supplement the distribution map of ecosystem types.”*

**Response4:**

The distribution map of ecosystem types was added in line 185(Figure 2) in marked-up copy of my manuscript. The modified part is highlighted in yellow.

**Comment5:**

*“from 2000-2015, what does this mean? The authors have to state clearly which year was used. ”*

**Response5:**

The mean is “from 2000, 2005, 2010, and 2015”. line 194-195 in marked-up copy of my manuscript. The modified part is highlighted in yellow.

**Comment6:**

*“Classification of ecosystem service functions. Please explain the specific problem of repeatability. The description is not fully understood due to the ecological integrity has been evaluated in previous research. ”*

**Response6:**

“The specific problem of repeatability” means is “the double- counting problem between ecological integrity and other ecosystem services”. line 217-219 in marked-up copy of my manuscript. The modified part is highlighted in yellow.

**Comment7:**

*“In the Table3, the unit of area is km2, while in formula 19, the unit of area is ha. ”*

**Response7:**

The unit of area is unified as km2. line 593 in marked-up copy of my manuscript. The modified part is highlighted in yellow.

**Comment8:**

*“Biodiversity maintenance value regulation index (a11), How the habitat quality index is measured? ”*

**Response8:**

The habitat quality index was measured in detail in another article of the author, so we added the reference. line 562-563 in marked-up copy of my manuscript. The modified part is highlighted in yellow.

**Comment9:**

*“"invest" should be "InVEST"”*

Response9:

We modified the invest to InVEST. line 562 in marked-up copy of my manuscript. The modified part is highlighted in yellow.

**Comment10:**

*“English needs proofreading and editing, especially those sentences related to comparison. In addition, there exists many formatting problems in this manuscript, please verify the full text. ”*

**Response10:**

We used Editage for English language editing that edited my manuscript, and we have carefully and thoroughly proofread the manuscript to correct all the grammar and typos. For details of manuscript editing, please refer to marked-up copy of my manuscript.

**Comment11:**

*“Please unify the number of decimal points in the whole text. ”*

**Response11:**

We unified the number of decimal points in the whole text.line 37, 42,567,612,632,646 in marked-up copy of my manuscript. The modified part is highlighted in yellow.

**Comment 12:**

*“Table1. Please note that the black space. ”*

**Response12:**

We revised the black space. line 314 in marked-up copy of my manuscript. The modified part is highlighted in yellow.

Reply to Reviewer #3

**Comment1:**

*“The paper takes Gansu Province as an example, on the basis of fully considering the 6 regional differences of ecosystem service function. But it don’t explain why Gansu was taken as an example? Does Gansu have a typical ecological service system? ”*

**Response1:**

We stated the reasons that the types of ecosystems are complex and diverse in Gansu Province, line 119-127 in marked-up copy of my manuscript. The modified part is highlighted in yellow.

**Comment2:**

*“making dialogue with the ESV evaluation and calculation literatures by taking the role of your specific context. ”*

**Response2:**

We have supplemented the relevant references on the other ecosystem service value studies, line108-118, 858-863 in marked-up copy of my manuscript. The modified part is highlighted in yellow.

**Comment3:**

*“This paper did a poor job in readability, with many typo errors and unstructured sentences. The authors need a copy editor before next submission. ”*

**Response3:**

We used Editage for English language editing that edited my manuscript, and we have carefully and thoroughly proofread the manuscript to correct all the grammar and typos. For details of manuscript editing, please refer to marked-up copy of my manuscript.
